# Human IL-32θA94V mutant attenuates monocyte-endothelial adhesion by suppressing the expression of ICAM-1 and VCAM-1 via binding to cell surface receptor integrin αVβ3 and αVβ6 in TNF-α-stimulated HUVECs

**DOI:** 10.3389/fimmu.2023.1160301

**Published:** 2023-05-09

**Authors:** Jae-Young Park, Hyo-Min Park, Seonhwa Kim, Kyeong-Bae Jeon, Chae-Min Lim, Jin Tae Hong, Do-Young Yoon

**Affiliations:** ^1^ Department of Bioscience and Biotechnology, Konkuk University, Seoul, Republic of Korea; ^2^ College of Pharmacy & Medical Research Center, Chungbuk National University, Cheongju, Republic of Korea

**Keywords:** IL-32θ, integrins, Intercellular adhesion molecule-1, vascular cell adhesion molecule-1, human umbilical vein endothelial cells, monocyte-endothelial adhesion

## Abstract

Interleukin-32 (IL-32), first reported in 2005, and its isoforms have been the subject of numerous studies investigating their functions in virus infection, cancer, and inflammation. IL-32θ, one of the IL-32 isoforms, has been shown to modulate cancer development and inflammatory responses. A recent study identified an IL-32θ mutant with a cytosine to thymine replacement at position 281 in breast cancer tissues. It means that alanine was also replaced to valine at position 94 in amino acid sequence (A94V). In this study, we investigated the cell surface receptors of IL-32θA94V and evaluated their effect on human umbilical vein endothelial cells (HUVECs). Recombinant human IL-32θA94V was expressed, isolated, and purified using Ni-NTA and IL-32 mAb (KU32-52)-coupled agarose columns. We observed that IL-32θA94V could bind to the integrins αVβ3 and αVβ6, suggesting that integrins act as cell surface receptors for IL-32θA94V. IL-32θA94V significantly attenuated monocyte-endothelial adhesion by inhibiting the expression of Intercellular adhesion molecule-1 (ICAM-1) and vascular cell adhesion molecule-1 (VCAM-1) in tumor necrosis factor (TNF)-α-stimulated HUVECs. IL-32θA94V also reduced the TNF-α-induced phosphorylation of protein kinase B (AKT) and c-jun N-terminal kinases (JNK) by inhibiting phosphorylation of focal adhesion kinase (FAK). Additionally, IL-32θA94V regulated the nuclear translocation of nuclear factor kappa B (NF-κB) and activator protein 1 (AP-1), which are involved in ICAM-1 and VCAM-1 expression. Monocyte-endothelial adhesion mediated by ICAM-1 and VCAM-1 is an important early step in atherosclerosis, which is a major cause of cardiovascular disease. Our findings suggest that IL-32θA94V binds to the cell surface receptors, integrins αVβ3 and αVβ6, and attenuates monocyte-endothelial adhesion by suppressing the expression of ICAM-1 and VCAM-1 in TNF-α-stimulated HUVECs. These results demonstrate that IL-32θA94V can act as an anti-inflammatory cytokine in a chronic inflammatory disease such as atherosclerosis.

## 1 Introduction

IL-32, previously known as NK4, is expressed in activated human T cells and NK cells after stimulation by mitogen or IL-2. Previous studies showed that NK4 induced pro-inflammatory cytokines such as IL-8, TNF-α, and MIP-2 in several immune cells through the classical cytokine signaling pathway, but lacked sequence homology with other cytokines. Hence, it was renamed IL-32, a part of one of the interleukin families (1). Numerous studies have reported that IL-32 is associated with cancer growth and development, viral infections, as well as chronic inflammatory diseases such as Crohn’s disease, inflammatory bowel disease, and rheumatoid arthritis, which demonstrated the ability of IL-32 to function as a cytokine (2–12). The IL-32 gene contains eight exons, with several splice variants differing in structure. IL-32α, IL-32β, IL-32γ, and IL-32δ were first discovered in NK cells, with IL-32γ having the longest sequence among the IL-32 isoforms (1). IL-32ε, IL-32ζ, IL-32η, IL-32θ, and IL-32sm were additionally identified, and a total of nine isoforms have been reported (13, 14). Early studies on IL-32 suggest that it is a pro-inflammatory cytokine. However, further studies on the individual isoforms show that they play different roles (15).

IL-32θ, one of the IL-32 isoforms, is the only isoform with an exon 6 deletion, except for IL-32sm. Recent studies have shown that IL-32θ has tumor suppression and anti-inflammatory effects (16, 17). We recently identified mutations in IL-32θ in tissues from patients with breast cancer. Sequence analyses revealed a cytosine to thymine replacement at position 281 in the mutant isoform, leading to a change in the amino acid sequence from alanine to valine at position 94 in the protein sequence. The mutant showed anti-inflammatory function inhibiting pro-inflammatory cytokines such as IL-1β, IL-6, IL-8, and COX2 in breast cancer cells (18). Previous studies on IL-32θA94V were limited to overexpression, and its specific receptor was not clearly identified. IL-32α and IL-32β have been reported to bind to integrin αVβ3 and integrin αVβ6 (19). In this study, we investigated whether these integrins could also act as receptors for IL-32θA94V.

Intercellular adhesion molecule-1 (ICAM-1) and vascular cell adhesion molecule-1 (VCAM-1) are markers of inflammatory responses involved in various diseases, including asthma and rheumatoid arthritis (20–25). Pro-inflammatory cytokines such as IL-1β and TNF-α (26, 27) induce the expression of ICAM-1 and VCAM-1 in vascular endothelial cells (26, 27). One of their functions is to initiate trans-endothelial migration (TEM) by arresting monocytes and leukocytes. TEM refers to the process that immune cells adhere to vascular endothelial cells and pass between them to migrate to the inflammation region. This process contribute to enhancement of the inflammatory response (28). ICAM-1 and VCAM-1 bind to lymphocyte function-associated antigen 1 (LFA-1) and very late antigen-4 (VLA-4) expressed in monocytes, triggering TEM by arresting the immune cells on the vascular endothelial cells (29). TEM is also important early stage of atherosclerosis. Atherosclerosis is characterized by inflammation, injured endothelial cells, and the formation of plaques due to the accumulation of oxidized low-density lipoproteins (ox-LDL) (30). Plaques contain vascular endothelial cells, smooth muscle cells, and lipid-containing macrophages known as foam cells. ICAM-1 and VCAM-1 induce TEM of monocytes, and the migrated monocytes develop plaques through uptake of ox-LDL (31, 32). Plaque development obstructs blood flow in vessels in various parts of the body, resulting in fatal disease such as cardiovascular disease (CVD) (33, 34). Therefore, regulation of ICAM-1 and VCAM-1 could be an important target for the treatment of various diseases as well as vascular inflammation (35).

Previous studies have suggested that IL-32β and IL-32γ act as pro-inflammatory cytokines involved in the upregulation of ICAM-1 and VCAM-1 (36, 37). In this study, we identified the cell surface receptors for IL-32θA94V and showed that IL-32θA94V was involved in the expression of ICAM-1 and VCAM-1, similar to other isoforms. Interestingly, IL-32θA94V downregulated the expression of ICAM-1 and VCAM-1 in contrast to other isoforms such as IL-32β and IL-32γ (36, 37). These findings support the differential roles of IL-32 isoforms and demonstrate the therapeutic potential of IL-32θA94V in inflammatory diseases such as atherosclerosis.

## 2 Materials and methods

### 2.1 Expression of human IL-32θA94V

IL-32θA94V coding sequence was synthesized by Bioneer (Daejeon, Korea) using HT-oligo™ synthesis. The synthesized DNA sequence was cloned into a pTH24 based TEVSH vector (Addgene, Watertown, MA, USA) using NdeI and AgeI restriction sites and rapid DNA ligation kit (Thermo Fisher Scientific, Waltham, MA, USA). TEVSH was a gift from Helena Berglund (Addgene plasmid # 125194; http://n2t.net/addgene:125194; RRID : Addgene 125194) and confirmed by DNA sequencing (Bionics, Seoul, Korea). The recombinant TEVSH vectors were transformed into DH5α by heat shock and purified using a mini prep kit (Intron Biotechnology, Sungnam, Korea). The IL-32θA94V expression vectors were transformed into Rosetta strain of *Escherichia coli* by heat shock transformation. Successfully transformed single colony was picked up and cultured at 37°C for 4 h in a 200-rpm incubator in Luria–Bertani (LB) media with ampicillin (100 μg/mL). The cultured mixture was transferred to 2.4 L of fresh LB media with ampicillin (100 μg/mL) and grown in a 200-rpm incubator at 37°C until it reached an OD_600_ of 0.6. IL-32θA94V was induced by adding 0.5 mM IPTG. Cells were grown in a 200-rpm incubator at 16°C for 16 h.

### 2.2 Purification of IL-32θA94V using Ni-NTA and CNBr-activated sepharose 4B columns

After 16 h, the cells were harvested by centrifugation at 8,000 rpm for 10 min at 4°C. The supernatant was discarded, and the pellet was lysed using lysis buffer (50 mM Tris-HCl pH 8.0, 10% glycerol, 0.1% Triton X-100, 1X protease inhibitor cocktail, 2 mM MgCl_2_, 0.1 mg lysozyme) and incubated on ice for 30 min. The lysed cells were sonicated (amplitude 35%, turn on 10 s, turn off 10 s) for 1 min using a sonicator (Sonics & Materials, Inc., Newtown, CT, USA). The lysed cells were centrifuged at 13,000 rpm for 30 min at 4°C, and the supernatant was collected. Ni-NTA resin (Thermo Fisher Scientific) was loaded into a PD-10 column and balanced with equilibration buffer (20 mM Tris-HCl pH 8.0, 200 mM NaCl). The lysate was mixed with 10 mL of equilibration buffer and loaded into the column and washed by washing buffer (20 mM Tris-HCl pH 8.0, 200 mM NaCl, 25 mM imidazole). After washing, elution buffer (20 mM Tris-HCl pH 8.0, 500 mM NaCl, 500 mM imidazole) was loaded into the column and the flow-through buffer containing IL-32θA94V was reloaded into IL-32mAb (KU32-52)-coupled CNBr-activated Sepharose 4B column that was prepared using CNBr-activated Sepharose 4B (Sigma-Aldrich, St. Louis, MO, United States) and monoclonal antibody IL-32 mAb KU32-52 (38). After several washing steps with Tris (pH 8.0), IL-32θA94V was eluted with 100 mM glycine (pH 3.0). The tube receiving the eluted solution contained 1 M of Tris (pH 8.0) with one-tenth of the total volume. The purified IL-32θA94V was dialyzed three times with phosphate-buffered saline (PBS) using spectra/Por membrane (Spectrum Laboratories, Piscataway, NJ, USA) for 2 h each. Absence of endotoxin was evaluated by using polymyxin B (Sigma-Aldrich).

### 2.3 3D structure modeling of IL-32θA94V and IL-32θA94V-integrin binding prediction

The tertiary structure of IL-32θA94V was analyzed by I-TASSER (Iterative Threading ASSEmbly Refinement), which is a hierarchical approach to protein structure prediction and structure-based function annotation. I-TASSER identifies structural templates with full-length models constructed by template-based fragment assembly simulations. Function insights of the target are then derived by re-threading the 3D models through protein function database. Protein-protein docking prediction was performed using the model with the highest confidence score among the models derived from I-TASSER (39–41). Integrin-IL-32θA94V binding was analyzed using HDOCK server (http://hdock.phys.hust.edu.cn), a protein-protein binding prediction tool. HDOCK predicts the binding complexes between two proteins using a hybrid docking strategy. Structures of extracellular segments of integrin αVβ3 (PDB ID: 1JV2) and αVβ6 (PDB ID: 5FFG) were provided by protein data bank (PDB). The docking scores are calculated by knowledge-based iterative scoring function ITScorePP or ITScorePR (42–46).

### 2.4 IL-32θA94V-integrin binding assay

MaxiSorp flat-bottomed 96-well plates (Nunc, Roskilde, Denmark) were coated with 1 μg/mL of recombinant αVβ3 and αVβ6 integrins (R&D Systems, Minneapolis, MN, USA) diluted in PBS and incubated overnight at 4°C. Next, the wells were blocked with 1% BSA (Invitrogen, Waltham, MA, USA) in PBS at 37°C for 1 h followed by three washing steps with PBS containing 0.05% Tween 20. Subsequently, some wells were preincubated with 10 μM cyclo-RGDfV peptide (Peptide Institute Inc, Osaka, Japan) or 10% fetal bovine serum (FBS), whereas others were incubated with PBS at 37°C for 1 h. Next, the wells were incubated with various concentrations of mutant IL-32θA94V diluted in PBS, with or without 10 μM cyclo-(RGDfV) or 10% FBS at 37°C for 1 h. The wells were washed three times with wash buffer and incubated with IL-32 mAb KU32-52 diluted in PBS at a concentration of 0.2 μg/mL at 25°C for 1 h. After the incubation, the wells were washed three times with wash buffer and incubated with mouse-IgGκ light chain binding protein conjugated to HRP (m-IgGκ BP-HRP) (BETHYL, Waltham, MA USA) at 25°C for 1 h. Next, the wells were washed three times with wash buffer and incubated with TMB substrate at 25°C for 20 min, and the color reaction was stopped with 2.5 N H_2_SO_4_. The absorbance/optical density was measured at 450 nm using a microplate reader (Apollo LB 9110, Berthold Technologies GmbH, Bad Wildbad, Germany).

### 2.5 Cell culture

HUVEC cells were cultured in Dulbecco modified Eagle medium (Welgene Incorporation, Daegu, Korea) supplemented with 10% (v/v) heat-inactivated fetal bovine serum (Hyclone Laboratories, Logan, UT, USA), penicillin (100 U/mL), and streptomycin (100 μg/mL). The cells were incubated in a 5% CO_2_-containing chamber at 37°C.

### 2.6 RNA extraction and reverse-transcription polymerase chain reaction (RT-PCR)

HUVEC cells (3 × 10^5^ cells/well) were incubated in 6-well plates for 24 h and starved for 4 h with serum-free media. Subsequently, the cells were pre-treated with IL-32θA94V (10 ng/mL) for 1 h and treated with TNF-α (10 ng/mL) for another 4 h. The treated cells were collected and lysed using the easy-BLUE™ Total RNA extraction kit (iNtRon Biotechnology, Seoul, South Korea) according to the manufacturer’s instructions. For reverse-transcription (RT) polymerase chain reaction (PCR), RNA (1 µg) was reverse-transcribed to cDNA using oligo (dT) primers and M-MuLV reverse transcriptase (New England Biolabs, Ipswich, MA, USA). The synthesized cDNA was amplified using a PCR Thermal Cycler Dice instrument (Takara, Otsu, Shiga, Japan). The following sets of primers were used: integrin αV, 5′-AGGAGAAGGTGCCTACGAAGCT-3′ (forward) and 5′-GCA CAGGAAAGTCTTGCTAAGGC-3′ (reverse); integrin β3, 5′-CATGGATTCCAGCAATGTCCTC C-3′ (forward) and 5′-TTGAGGCAGGTGGCATTGAAGG-3′ (reverse); integrin β6, 5′- TCTCCTGCGTGAGACACAAAGG-3′ (forward), and 5′-GAGCACTCCATCTTCAGAGACG-3′ (reverse); ICAM-1, 5′-AGCGGCTGACGTGTGCAGTAAT-3′ (forward), 5′- TCTGAGACCTCTGGCTTCGTCA-3′ (reverse); VCAM-1,5′-GATTCTGTGCCCACAGTAAGG C-3′ (forward) and 5′-TGGTCACAGAGC CACCTTCTTG-3′ (reverse); glyceraldehyde 3-phosphate dehydrogenase (GAPDH), 5′-AGAACATCATCCCTGCCTCT-3′ (forward), 5′-CTGCTT CACCACCTTCTTGA-3′ (reverse). GAPDH was used as an internal control. The PCR products were separated on a 2% agarose gel.

### 2.7 Reverse transcription-quantitative polymerase chain reaction (RT-qPCR)

HUVECs were harvested after treatment with or without IL-32θA94V and TNF-α. mRNA extraction and cDNA synthesis were performed as described above. RT-PCR was performed with a relative quantification protocol using Rotor-Gene 6000 series software 1.7 (Qiagen, Hilden, Germany) and the Sensi FAST™ SYBR NO-ROX Kit (BIOLINE, London, UK). The expression of all target genes was normalized to that of the housekeeping gene, GAPDH. Each sample was run with the following primer sets: ICAM-1, 5′-AGCGGCTGACGTGTGCAGTAAT-3′ (forward), 5′- TCTGAGACCTCT GGCTTCGTCA-3′ (reverse); VCAM-1,5′-GATTCTGTGCCCACAGTAAGG C-3′ (forward), 5′-TGGTCACAGAGCCACCTTCTTG-3′ (reverse); GAPDH, 5′-AGAACATCATCCCTGCCTCT-3′ (forward), 5′-CTGCTTCACCACCTTCTTGA-3′ (reverse). GAPDH was used as an internal control. The mRNA levels of each gene were calculated relative to that of the internal reference, GAPDH using the comparative Ct method (47).

### 2.8 Immunoblotting

HUVEC cells (3 × 10^5^ cells/well) were seeded in 6-well plates for 24 h and pre-treated for 1 h with IL-32θA94V (100 ng/mL), followed by treatment with TNF-α (10 ng/mL). The cells were lysed in a buffer containing 50 mM Tris (pH 7.4), 150 mM NaCl, 1% NP-40, 0.1% sodium dodecyl sulfate (SDS), 0.25% sodium deoxycholate, 1 mM ethylene diamine tetraacetic acid (EDTA), 1 mM ethylene glycol tetraacetic acid, 1 mM orthovanadate, aprotinin (10 µg/mL), and 0.4 mM phenyl methylsulfonyl fluoride (PMSF) at 4°C for 2 h. The cells were lysed, and the protein content was estimated using a Bradford assay reagent (Bio-Rad Laboratories, Hercules, CA, USA) and UV spectrophotometer (48). The proteins (30 µg) were separated on 10% SDS-polyacrylamide gels and transferred to PVDF membranes. The membranes were blocked with 5% skim milk for 1 h at 25°C and incubated with primary antibodies against IL-32, His-tag (Sigma-Aldrich), FAK (Thermo Fisher Scientific), AKT (Cell Signaling, Danvers, MA, USA), JNK (Cell Signaling), IκB (Cell Signaling), p65 (Cell Signaling), p50 (Cell Signaling), PARP (Cell Signaling), c-Jun (Santa Cruz Biotechnology, Dallas, TX, USA), and c-Fos (Santa Cruz Biotechnology), for 1 h at 25°C. After incubation, the membranes were incubated with secondary antibodies (anti-rabbit or anti-mouse IgG antibodies) (BETHYL) for 1 h at 25°C. Finally, the protein bands were visualized using an enhanced chemiluminescence Western blotting detection kit (Advansta, San Jose, CA USA).

### 2.9 Immunofluorescence

HUVEC cells (1.0 × 10^5^ cells/well) were seeded in an 8-well slide chamber for 24 h and starved overnight, followed by pre-treatment for 1 h with IL-32θA94V (100 ng/mL) and a 6 h treatment with TNF-α (10 ng/mL). After the treatment, cells were fixed using 4% paraformaldehyde for 10 min followed by incubation with ice-cold methanol and blocking using 1% BSA in PBS for 1 h at 25°C. The cells were then incubated with primary antibodies against ICAM-1 and VCAM-1 (Beijing Solarbio Science & Technology Co., Beijing, China) overnight at 4°C, and with secondary antibodies conjugated to Cy3 (Merck Millipore, Darmstadt, Germany) for 1 h at 25°C, followed by DAPI staining. Two washing steps with PBS were performed between each step. Thereafter, cells were mounted in mounting buffer (Sigma-Aldrich) and examined under a fluorescence microscope.

### 2.10 siRNA transfection

HUVEC cells (3 × 10^5^ cells/well) were seeded in 6-well plates for 24 h and changed to fresh media. For silencing ICAM-1 and VCAM-1 expression, cells were transfected with the following siRNA sets (Bionics, Seoul, Korea): NC, 5′-UUCUCCGAACGUGUCACGUTT-3′; ICAM-1, 5′-UUCUUGUGUAUAAGCUGGCCGTT-3′; VCAM-1, 5′-CCAUUGUUCUCAUGGAGAATT-3′; (49, 50). INTERFERin reagent (Polyplus, Illkirch, France) was used for transfection, according to the manufacturer’s instruction. The final siRNA concentrations were 1 nM (VCAM-1) and 5 nM (ICAM-1), respectively. After 24 h of transfection, the cells were starved for 4 h followed by treatment with TNF-α (10 ng/mL) for another 4 h. The silencing efficiency was measured by RT-PCR analysis.

### 2.11 Monocyte-endothelial cell adhesion assay

HUVEC cells (1.5 × 10^5^ cells/well) were seeded in a 4-well slide chamber for 24 h and starved overnight, pre-treated with IL-32θA94V (100 ng/mL) for 1 h, followed by 6 h treatment with TNF-α (10 ng/mL). The THP-1 cells were labeled with 5 µM calcein-AM (Molecular Probes, Eugene, OR, USA) for 30 min in RPMI-1640 without FBS. The THP-1 cells labeled with calcein-AM were added to the 4-well slide chamber containing the HUVECs and incubated for 30 min in RPMI-1640 containing 10% FBS. Subsequently, unbound monocytes were removed by three washes with PBS. Remaining monocytes were determined using a fluorescence microscope. The intensity of fluorescence was measured using ImageJ software version 1.5 (51).

### 2.12 Preparation of cytosol and nuclear extracts

HUVEC cells (2.5 × 10^5^ cells/dish) were seeded in a cell culture dish for 24 h, pre-treated with IL-32θA94V (100 ng/mL) for 1 h, and treated with TNF-α (10 ng/mL) for 30 min before harvesting and fractionating using NE-PER nuclear and cytoplasmic extraction reagents (Thermo Fisher Scientific) according to the manufacturer’s instructions. Equal quantities of protein from these extracts (50 μg) were separated by SDS-polyacrylamide gel electrophoresis and transferred to PVDF membranes. The subsequent steps for the procedure were followed as described for Western blotting above. PARP was used as a nuclear protein marker.

## 3 Results

### 3.1 Expression and purification of recombinant human IL-32θA94V

**Figure 1:**
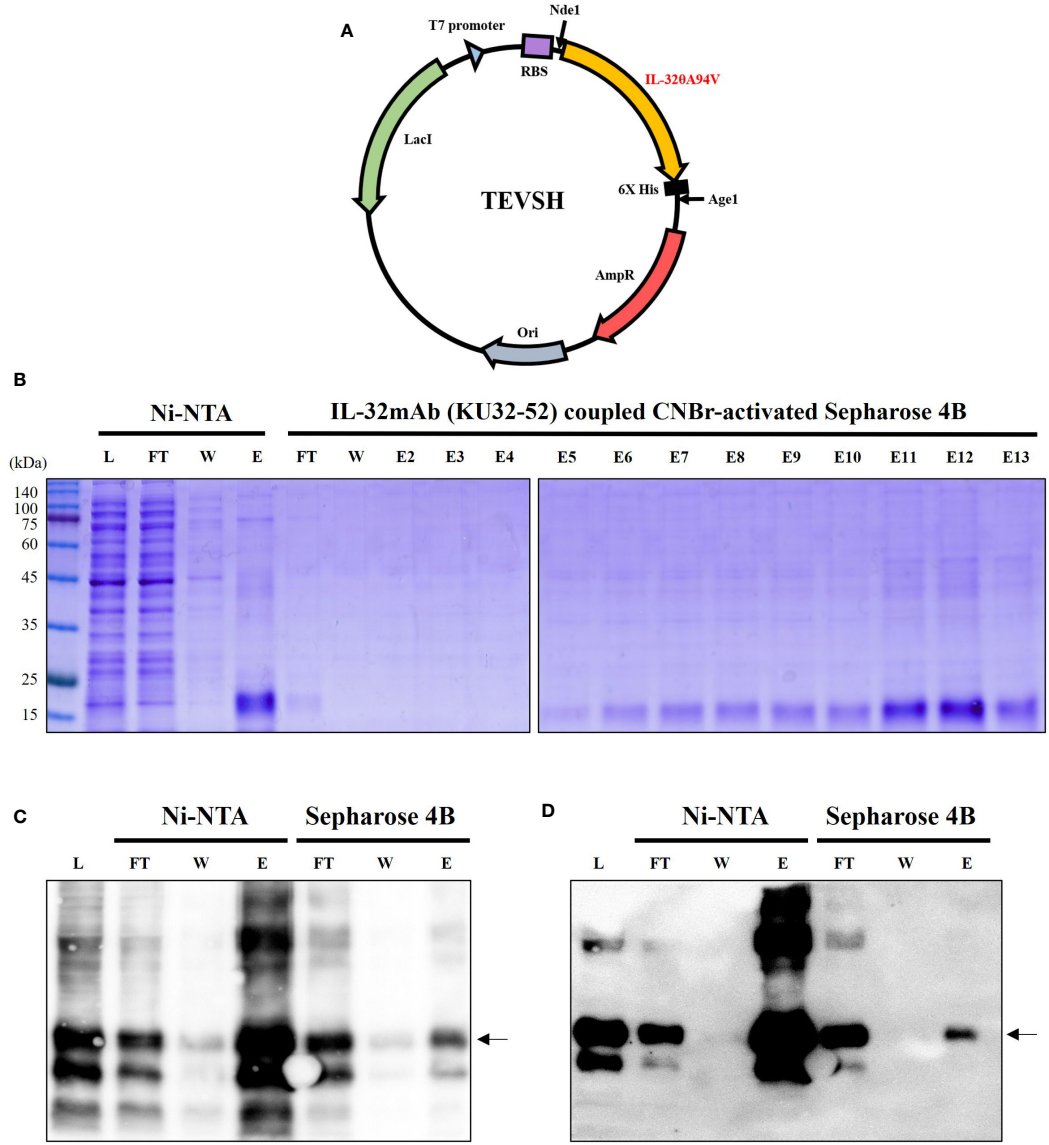
Expression and purification of IL-32θA94V. **(A)** Schematic diagram of mutant IL-32θA94V expression vector. As a protein expression vector, TEVSH with pTH24 as a backbone was used. His tag was included for purification. Nde1, Age1 restriction enzymes were used to construct recombinant DNA. SDS-PAGE **(B)** and Western blot using KU32-52 mAb **(C)** or anti-His tag mAb **(D)** were performed to confirm the isolated protein. IL-32θA94V was purified twice by Ni-NTA column followed by IL-32mAb (KU32-52) coupled CNBr-activated Sepharose 4B. IL-32θA94V bound to Ni-NTA column was eluted with 500 mM imidazole, and further purification was performed by IL-32 mAb (KU32-52)-coupled CNBr-activated Sepharose 4B using low pH Tris-glycine buffer (pH 3.0). L, lysate of *Escherichia coli* harboring IL-32θA94V expression vector; FT, flow through; W, washing flow; E, elution; E2-E13, eluted using low pH Tris-glycine buffer (pH 3.0).

TEVSH vector was used for expression of IL-32θA94V, and recombinant IL-32θA94V DNA was inserted into the vector by Nde1 and Age1 restriction enzymes. The TEVSH vector contained a His tag for protein purification. A schematic diagram of the recombinant vector is shown in **Figure 1A**. We performed consecutive purifications to improve the purity of IL-32θA94V. First, IL-32θA94V was separated by binding on a Ni-NTA column and His tag, and the separated IL-32θA94V was purified once more by a CNBr-activated Sepharose 4B column coupled with IL-32mAb KU32. Purified IL-32θA94V was analyzed using SDS-PAGE (**Figure 1B**) and Western blots (**Figures 1C, D**). In SDS-PAGE analysis, no other notable protein bands were detected except for IL-32θA94V in the eluate of the Ni-NTA column, but several other proteins were observed using Western blotting. These non-target proteins were removed by further purification using CNBr-activated Sepharose 4B column coupled to KU32-52. In the end, highly pure IL-32θA94V protein was obtained with few other proteins.

### 3.2 Binding of IL-32θA94V to integrin αVβ3 and αVβ6

**Figure 2:**
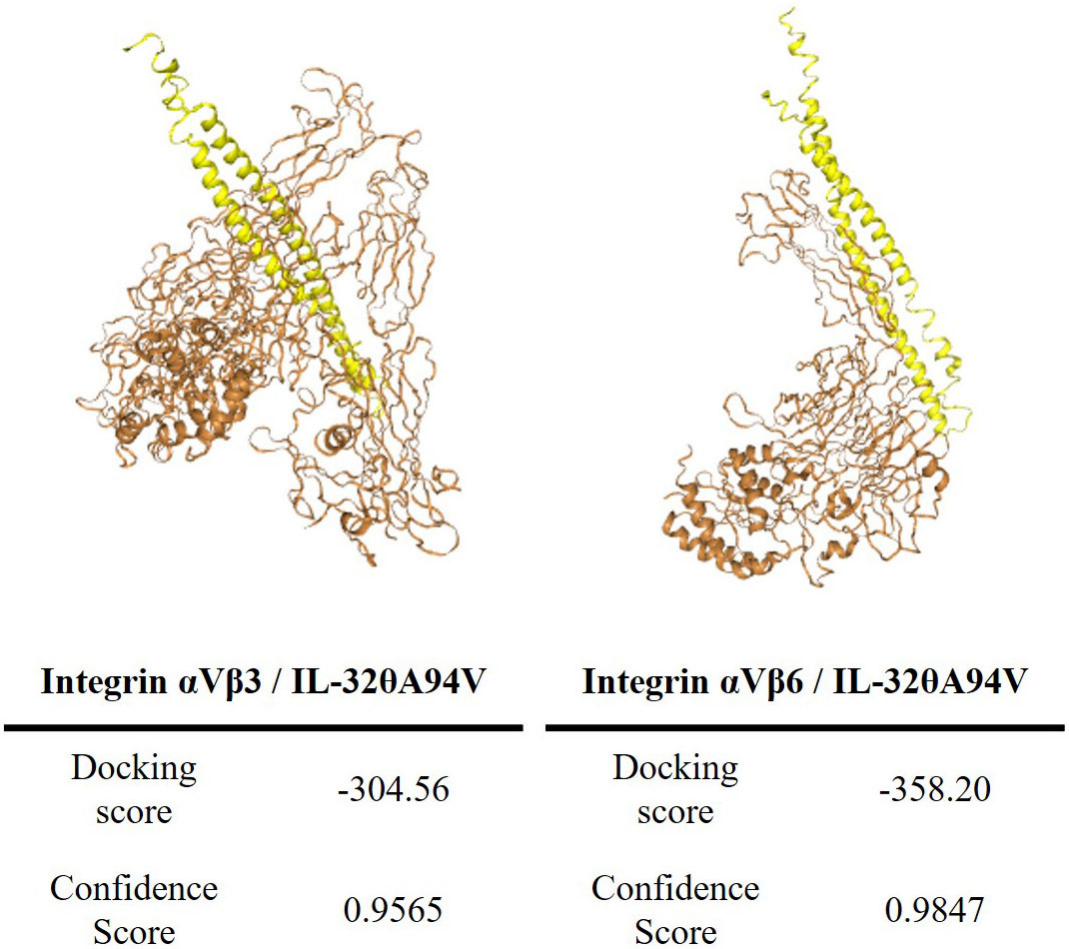
The tertiary structure modeling of IL-32θA94V and prediction of IL-32θA94V-integrin binding. Protein-protein associations were predicted by the HDOCK server (IL-32θA94V: yellow, integrins: orange). The tertiary structure of IL-32θA94V was constructed by I-TASSER, and the integrin was provided by PDB.

We predicted the binding of IL-32θA94V to integrins using the HDOCK server. IL-32θA94V was indicated as a yellow molecule containing a helix structure, and the extracellular domain of integrin αVβ3 and αVβ6 provided from PDB were indicated as orange molecules. The binding score of IL-32θA94V-integrin αVβ3 was -304.56, and the confidence score was 0.9565. The binding score of IL-32θA94V-integrin αVβ6 was -358.20, and the confidence score was 0.9847, which was higher than that of IL-32θA94V-integrin αVβ3 (**Figure 2**).

**Figure 3:**
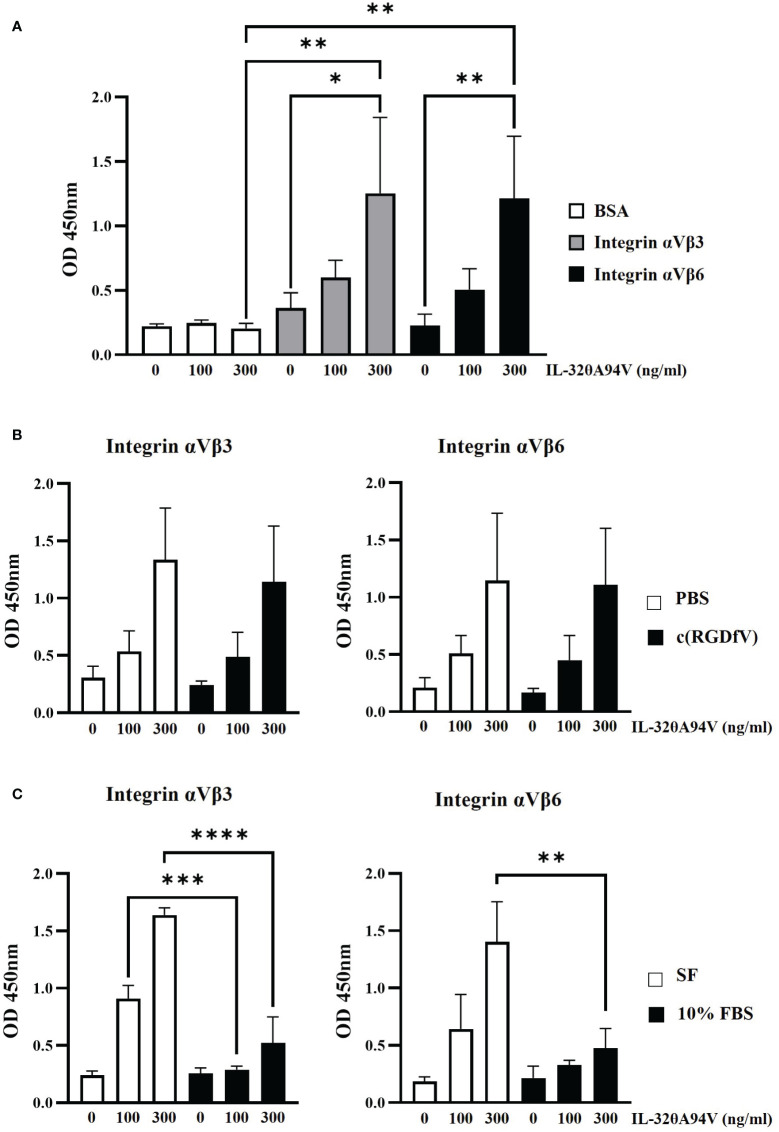
IL-32θA94V binding to recombinant αVβ3 and αVβ6 integrins. **(A)** IL-32θA94V binds to the recombinant αVβ3 and αVβ6 integrins. The effects of cyclo-(RGDfV) **(B)** and FBS **(C)** on the bindings between IL-32θA94V and recombinant αVβ3 or αVβ6 integrins. The bindings between IL-32θA94V and recombinant αVβ3 or αVβ6 integrins were assessed using IL-32 mAb (KU32-52). The results represent the mean ± SD of three experiments (*p < 0.05, ** p < 0.01, *** p < 0.001, **** p < 0.0001 by one-way ANOVA).

In addition to structure-based binding prediction (**Figure 2**), we also performed an IL-32θA94V-integrin binding assay (**Figure 3**). After coating 96-well plates with integrin αVβ3 and αVβ6, IL-32θA94V was added at the concentration shown in **Figure 3A**. In the presence of the integrins, the absorbance increased in a concentration-dependent manner of IL-32θA94V but not in BSA (**Figure 3A**), indicating that IL-32θA94V also binds to the integrins αVβ3 and αVβ6 similar to other isoforms. Cyclo-RGDfV, which is known to bind to the RGD-binding site of integrins, was pretreated in 96-well plates, and PBS was used as a control. Further, wells were coated with integrins αVβ3 and αVβ6. IL-32θA94V was treated at the concentrations shown in **Figure 3B**. In both integrins αVβ3 and αVβ6, integrin-IL-32θA94V binding was not significantly reduced by cyclo-(RGDfV) (**Figure 3B**), suggesting that IL-32θA94V binds to the non-RGD binding sites. In the other group, media containing 10% FBS were pretreated in 96-well plates. Integrins αVβ3 and αVβ6 were then added, and the control group was pretreated with serum-free media. IL-32θA94V was treated at the concentrations shown in **Figure 3C**. In contrast to cyclo-(RGDfV), integrin-IL-32θA94V binding was significantly inhibited by FBS (**Figure 3C**). These results demonstrated that IL-32θA94V binds to the non-RGD binding site of integrin αVβ3 and αVβ6, similar to other isoforms such as IL-32β, and suggest that these integrins can act as cell surface receptors for IL-32θA94V.

### 3.3 Effects of IL-32θA94V on expression of ICAM-1 and VCAM-1 in TNF-α-stimulated HUVEC cells

**Figure 4:**
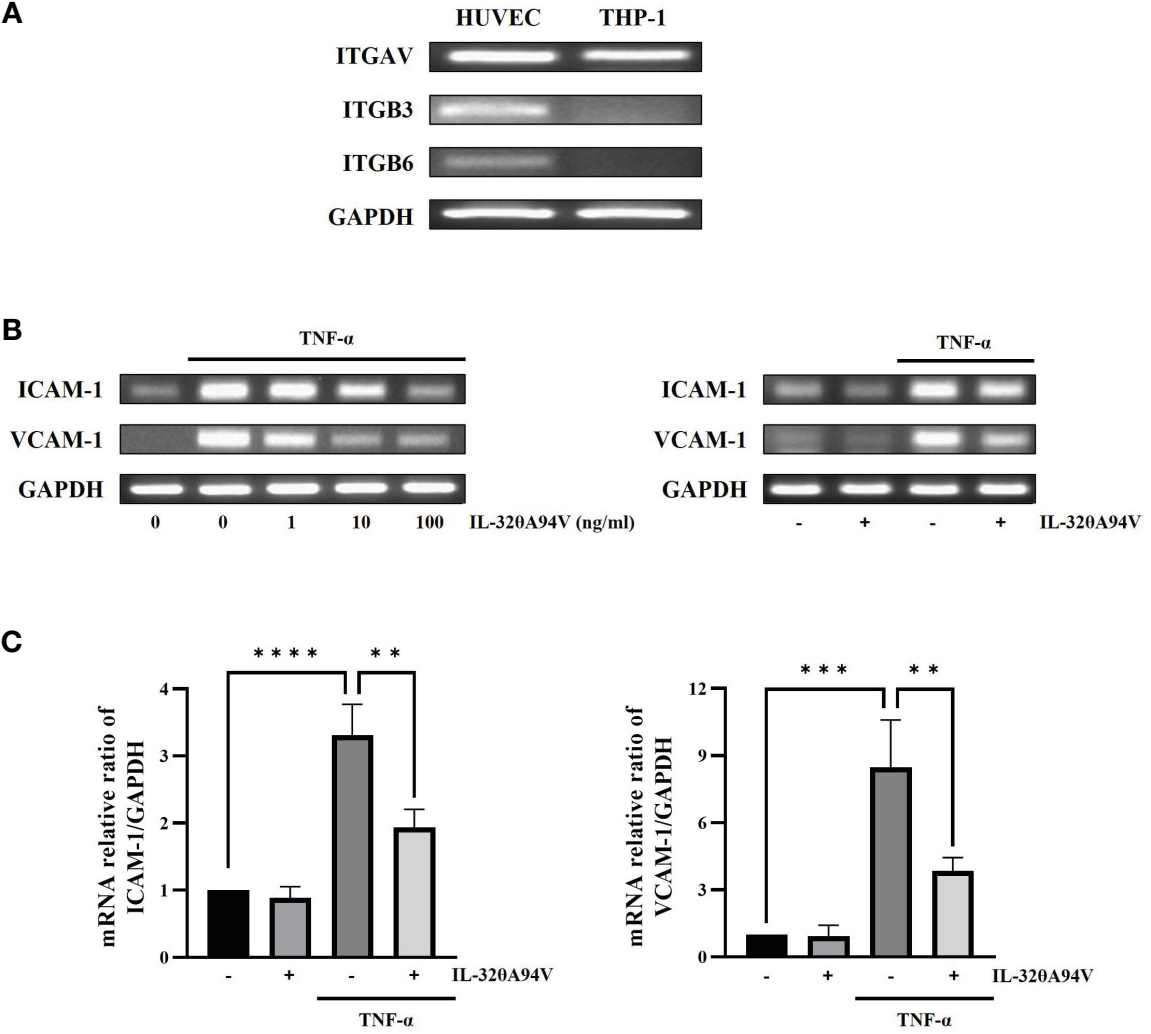
The effect of IL-32θA94V on mRNA expression of ICAM-1 and VCAM-1 in TNF-α stimulated human umbilical vein endothelial cells (HUVECs). **(A)** The expression of integrins αV, β3 and β6 subunits were confirmed by RT-PCR in HUVEC cells but not in THP-1 human monocytic cells. HUVECs were pre-treated with IL-32θA94V (100 ng/mL) for 1 h and stimulated with TNF-α (10 ng/mL) for 4 h. mRNA expression of adhesion molecules was detected by RT-PCR **(B)** and RT-qPCR **(C)** analyses. The results represent the mean ± SD of three experiments (** p < 0.01, *** p < 0.001, **** p < 0.0001 by one-way ANOVA).

**Figure 5:**
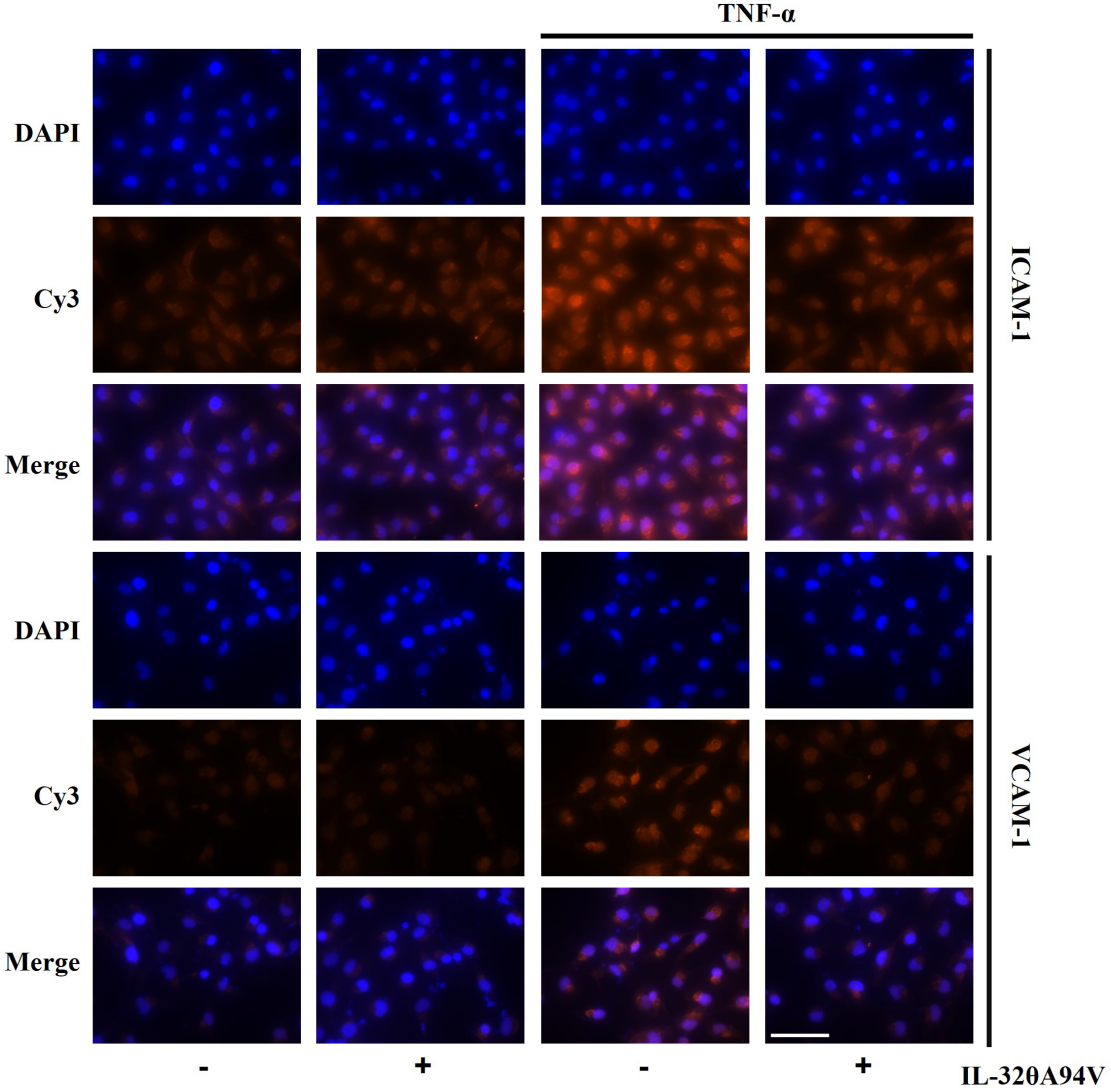
The effect of IL-32θA94V on protein expression of ICAM-1 and VCAM-1 in TNF-α stimulated human umbilical vein endothelial cells (HUVECs). HUVECs were pre-treated with IL-32θA94V (100 ng/mL) for 1 h and stimulated with TNF-α (10 ng/mL) for 6 h. Protein expression of adhesion molecules was analyzed by immunofluorescence using specific antibodies. Scale bar in each image represents 75 μm.

We confirmed the expression of integrins in HUVECs to evaluate the effects of IL-32θA94V on cells. THP-1 monocytes were used as a negative control, which did not express integrin αVβ3 and αVβ6 on their surfaces (**Figure 4A**). And, we investigated the effect of IL-32θA94V on the expression of ICAM-1 and VCAM-1 in TNF-α-stimulated HUVEC cells. HUVECs were starved for 4 h to minimize the interference by FBS, and IL-32θA94V was pretreated for 1 h and stimulated with TNF-α for 4 h or 6 h. The mRNA levels of ICAM-1 and VCAM-1 were measured using RT-PCR (**Figure 4B**) and RT-qPCR (**Figure 4C**). mRNA expression levels of ICAM-1 and VCAM-1 were increased by TNF-α and significantly decreased by IL-32θA94V. The protein expressions were also analyzed using immunofluorescence. The fluorescence intensities of ICAM-1 and VCAM-1 were increased upon TNF-α stimulation and suppressed by IL-32θA94V as expected (**Figure 5**).

### 3.4 Effects of IL-32θA94V on monocyte-endothelial cell adhesion

**Figure 6:**
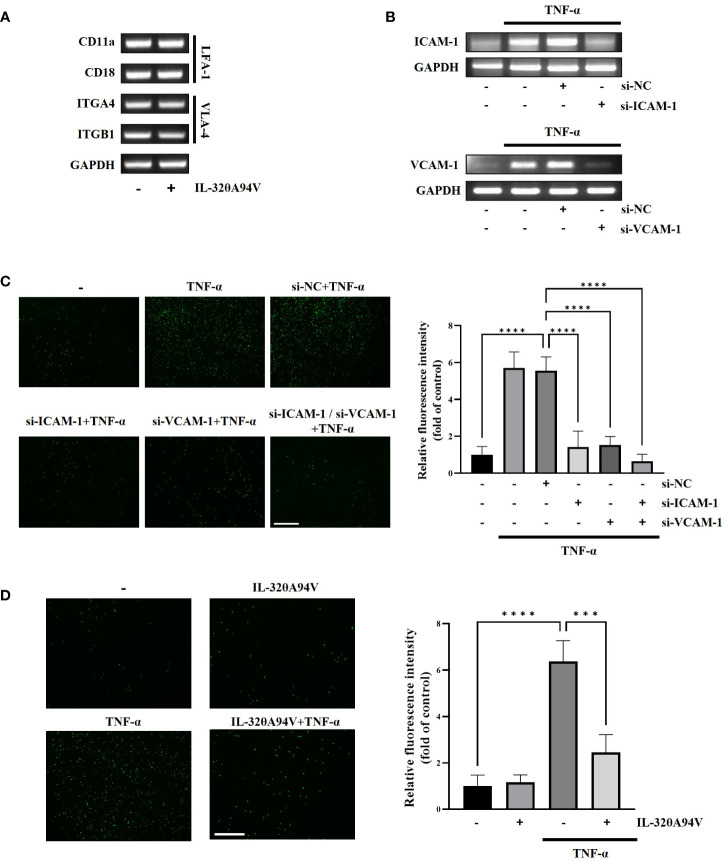
IL-32θA94V attenuates monocyte adhesion to HUVECs. **(A)** THP-1 cells were treated with IL-32θA94V (100 ng/ml) for 24 h. LFA-1 (CD11a/CD18) and VLA-4 (ITGA4/ITGB1) expression in THP-1 cells was confirmed by RT-PCR analysis. **(B)** HUVECs were transfected with siRNA for 24 h followed by starved 4 h, then, treated with TNF-α for 4 h. siRNA transfection efficiency was evaluated by RT-PCR analysis. **(C, D)** Fluorographs of calcein-AM-labeled THP-1 cell attachment to HUVECs. HUVECs were incubated with calcein-AM-labeled THP-1 for 30 min after treatment or transfection as shown in figure. Fluorescence was measured using ImageJ software. Quantified fluorescence intensity was normalized to the control. Scale bar in each image represents 650 μm. The results represent the mean ± SD of three experiments (*** p < 0.001, **** p < 0.0001 by one-way ANOVA).

As LFA-1 and VLA-4 are known to bind ICAM-1 and VCAM-1, respectively, we confirmed the expression of LFA-1 and VLA-4 in THP-1 cells. Expression of these molecules implies that THP-1 can attach to HUVECs through the interaction of ICAM-1and VCAM-1. Additionally, the expression of LFA-1 and VLA-4 was not reduced by treatment of IL-32θA94V (**Figure 6A**). We used siRNA transfection to verify whether downregulation of ICAM-1 and VCAM-1 leads to suppression of monocyte-endothelial adhesion. The mRNA levels of TNF-α-induced ICAM-1 and VCAM-1 were reduced by siRNA transfection (**Figure 6B**). In co-cultures of THP-1 cells and HUVECs, residual calcein-AM-labeled THP-1 (green) was increased by TNF-α and attenuated by siRNA transfection (**Figure 6C**). These results suggest that the monocyte-endothelial adhesion was regulated by the expression levels of ICAM-1 and VCAM-1. Previously, in **Figure 4** and **5**, we found that IL-32θA94V inhibited ICAM-1 and VCAM-1 expression. Thus, we investigated whether IL-32θA94V could attenuate monocyte-endothelial adhesion. Adhesion between calcein-AM-labeled THP-1 and HUVECs was increased by TNF-α and significantly suppressed by IL-32θA94V (**Figure 6D**). Taken together, it was suggested that IL-32θA94V attenuated monocyte-endothelial adhesion by inhibiting the expression of ICAM-1 and VCAM-1 in TNF-α-stimulated HUVECs.

### 3.5 Effects of IL-32θA94V on phosphorylation levels of FAK, AKT, and JNK in TNF-α-stimulated HUVEC cells

**Figure 7:**
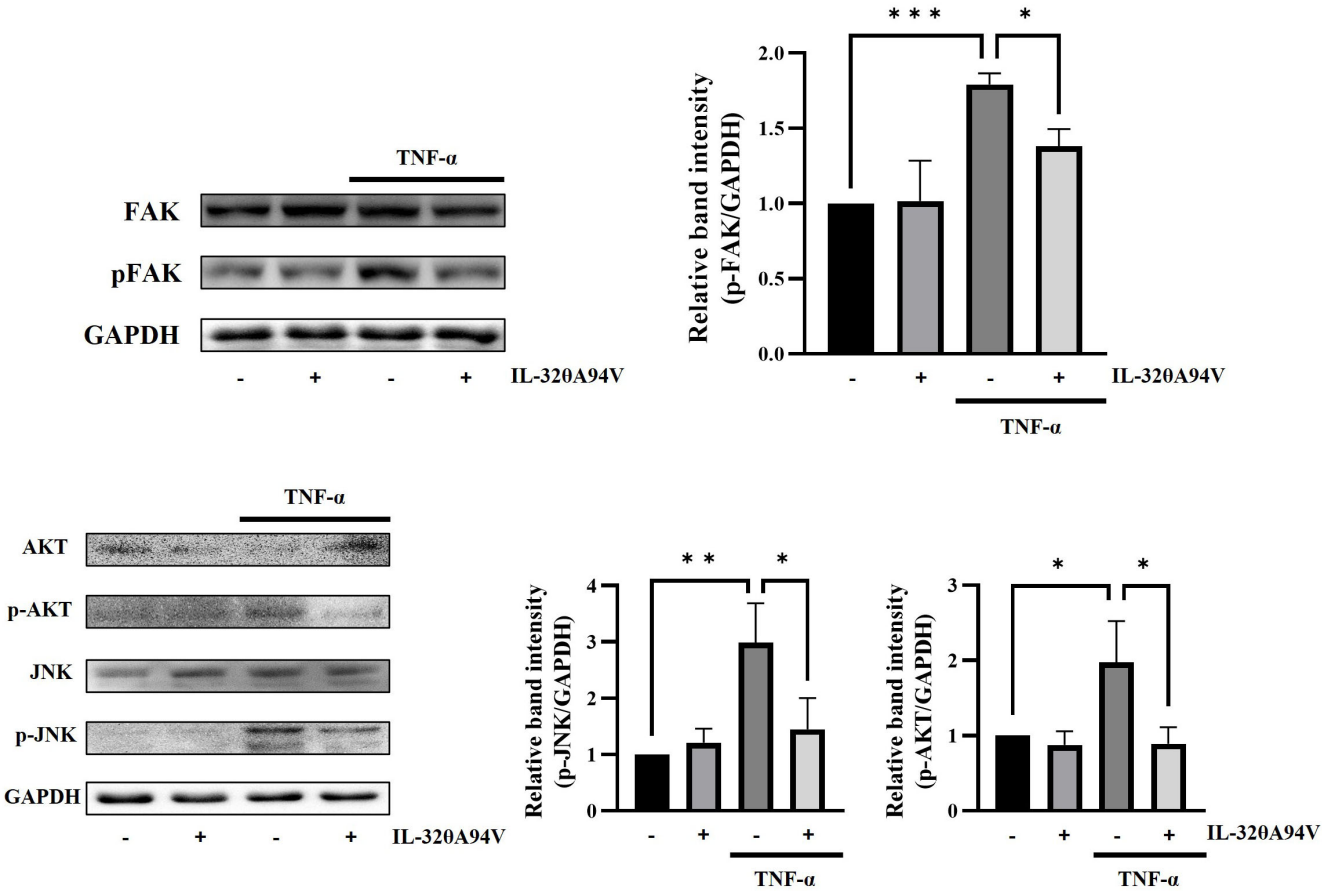
Effect of IL-32θA94V on phosphorylation level of FAK, JNK, and AKT in HUVEC cells. HUVECs were pre-treated with IL-32θA94V (100 ng/mL) for 1 h followed by stimulation with TNF-α for 10 min (FAK) or 15 min (JNK, AKT). Phosphorylation levels of FAK, AKT, and JNK were confirmed by Western blot, and band intensities were quantified using ImageJ software. The results represent the mean ± SD of three experiments (*p < 0.05, ** p < 0.01, *** p < 0.001 by one-way ANOVA).

We revealed that IL-32θA94V inhibited the expression of ICAM-1 and VCAM-1 in TNF-α-stimulated HUVECs. We performed Western blot to identify the intracellular signaling pathway underlying the regulation of ICAM-1 and VCAM-1 expression mediated by IL-32θA94V. IL-32θA94V significantly reduced the phosphorylation level of TNF-α-induced focal adhesion kinase (FAK), a well-known integrin-mediated signaling molecule, which is an upstream molecule of protein kinase B (AKT) and c-Jun N-terminal kinase (JNK). IL-32θA94V also downregulated phosphorylation levels of AKT and JNK by inhibiting FAK, as expected (**Figure 7**).

### 3.6 Effects of IL-32θA94V on nuclear translocation of NF-κB and AP-1 in TNF-α-stimulated HUVEC cells

**Figure 8:**
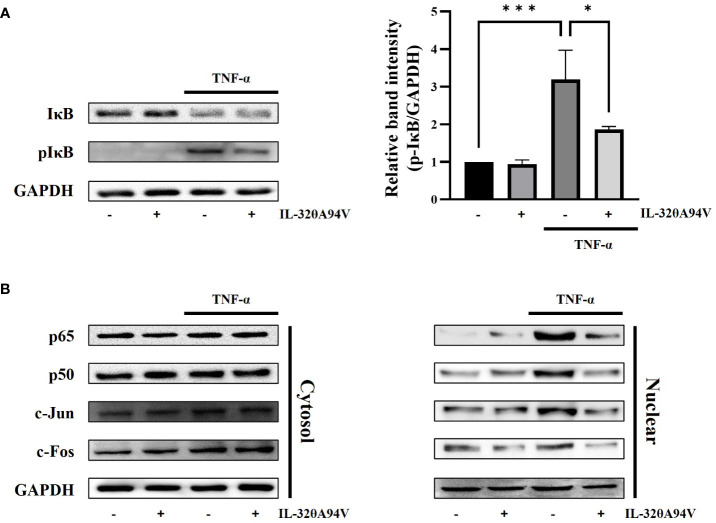
The effect of IL-32θA94V on phosphorylation levels of IκB and nuclear translocation of NF-κB (p65/p50), AP-1 (c-Fos/c-Jun) in HUVEC cells. HUVEC cells were incubated with IL-32θA94V for 1 h, then stimulated with TNF-α (10 ng/ml) for another 10 min (IκB) or 30 min (transcription factors). Harvested cells were subjected to nuclear fractionation. Phosphorylation and translocation levels were analyzed by Western blot, and band intensities were quantified using ImageJ software. The results represent the mean ± SD of three experiments (*p < 0.05, *** p < 0.001 by one-way ANOVA).

TNF-α stimulation activates the FAK/AKT signaling pathway, leading to nuclear translocation of NF-κB (p65/p50) via phosphorylation of IκB. In addition, activation of JNK accelerates nuclear translocation of AP-1 (c-fos/c-jun). These transcription factors promote the expression of ICAM-1 and VCAM-1 (52, 53). We confirmed the phosphorylation level of IκB and nuclear translocation of NF-κB (p65/p50) and AP-1 (c-Fos/c-Jun). IL-32θA94V marginally attenuated the TNF-α-induced IκB phosphorylation (**Figure 8A**). Nuclear translocation of AP-1 (c-Fos/c-Jun) and NF-κB (p65/p50) was increased by TNF-α stimulation and was inhibited by IL-32θA94V (**Figure 8B**). These results show that IL-32θA94V regulated the nuclear translocation of AP-1 (c-Fos/c-Jun) and NF-κB (p65/p50) in TNF-α-stimulated HUVECs, which in turn results in the attenuation of ICAM-1 and VCAM-1 expression.

## 4 Discussion

IL-32 is involved in various cell functions such as apoptosis, differentiation, viral infection, and modulation of inflammatory cytokines, indicating that it is a cytokine that plays key roles in several diseases. However, the role of IL-32 isoforms can vary depending on experimental conditions such as cell lines or diseases. Previous studies on IL-32 suggest the necessity to define the various functions of IL-32 isoforms. For example, IL-32α and IL-32β are cytokines with both pro- and anti-inflammatory properties (54–57). IL-32γ has mainly pro-inflammatory properties by inducing pro-inflammatory cytokines such as IL-6, IL-12, and CCL5 (58, 59). IL-32θ is known to have anti-inflammatory and tumor suppression properties (60, 61).

Recently, an IL-32θA94V mutant was discovered in the tissues from a patient with breast cancer, and IL-32θA94V was found to suppress the expression of pro-inflammatory cytokines in breast cancer cells (18). Wild type IL-32θ recombinant protein has been reported to have the most dominant biological activity among the seven IL-32 isoforms. It significantly increased IL-6, IL-8 and TNF-α in various cells (62). However, in this study, we purified a recombinant IL-32θA94V protein and investigated whether a IL-32θ mutant would possessed the anti-inflammatory effects mediated via cell surface receptors as an exogenous modulator.

We designed a recombinant vector using Nde1 and Age1 restriction enzymes (**Figure 1A**). The vector was transformed into *Rosetta*, one of the strains of *E. coli*, by the heat-shock method. The insert DNA contained a His-tag for purification. We obtained pure IL-32θA94V using sequential purification of Ni-NTA column and an IL-32 mAb KU32-52 coupled CNBr-activated Sepharose 4B column. Purified proteins were confirmed by SDS-PAGE (**Figure 1B**) and Western blot (**Figures 1C, D**).

Most IL-32 isoforms, including IL-32θA94V, have an RGD motif, and integrin αVβ3 and αVβ6 are known to bind to the RGD motif. In addition, it was reported that these integrins bind to IL-32α and IL-32β (19). Thus, we hypothesized that IL-32θA94V would also bind to these integrins, and analyzed using protein-protein binding prediction tool, HDOCK server. According to the description of the HDOCK server, protein-protein binding typically has a score of -200 or higher, more negative docking score means a more possible binding model. Also, confidence score of 0.7 or higher means that the two molecules are highly likely to bind at -200 or higher docking score (42–46). Therefore, the result of docking prediction suggests that IL-32θA94V has strong binding potential with integrin αVβ3 and integrin αVβ6. IL-32θA94V showed a higher docking score with integrin αVβ6 than integrin αVβ3 (**Figure 2**). However, no significant difference was identified in the IL-32θA94V-integrin binding assay. IL-32θA94V was found to bind to both integrin αVβ3 and αVβ6 in a dose dependent manner (**Figure 3A**), suggesting that these integrins can act as receptors for IL-32θA94V. However, the interaction was not blocked by cyclo-(RGDfV), including the short RGD peptide, indicating that, contrary to expectations, IL-32θA94V-integrin binding is not mediated by the RGD motif (**Figure 3B**). Extracellular matrix such as fibronectin, vitronectin, and growth factors included in FBS are known to bind to integrins (63–65). They can block an integrin-IL-32θA94V interaction by binding to integrins. We observed that the interaction was reduced in a 10% FBS-containing medium compared to that in the serum free media, as expected (**Figure 3C**).

RT-PCR analyses confirmed the expression of integrins αVβ3 and αVβ6 in HUVEC cells but not in THP-1 human monocytic cells. We also demonstrated that TNF-α-induced upregulation of ICAM-1 and VCAM-1 expression was significantly decreased in IL-32θA94V-pretreated HUVECs (**Figures 4**, **5**). These results show an opposite role of IL-32θA94V, compared to IL-32β and IL-32γ, which are known to induce the expression of these cell adhesion molecules (36, 37).

We confirmed that monocyte–endothelial adhesion was mediated by ICAM-1 and VCAM-1 using siRNA transfection (**Figures 6B, C**). Further, we investigated whether IL-32θA94V attenuated monocyte–endothelial adhesion by suppressing expression of these cell adhesion molecules. THP-1 monocytes were stained with the fluorescent dye calcein-AM and co-cultured with HUVECs. After several washes, the green staining fluorescence intensity of TNF-α-treated THP-1 cells was significantly enhanced, while fluorescence intensity of monocytes was reduced by IL-32θA94V (**Figure 6D**). These results demonstrated that IL-32θA94V attenuates monocyte endothelial adhesion by inhibiting the expression of cell adhesion molecules, ICAM-1 and VCAM-1, in TNF-α-stimulated HUVECs.

FAK is a well-known integrin-mediated signaling molecule, which is closely involved in intracellular signals induced by various cytokines and growth factors (66). Phosphorylated FAK induced by these molecules activates the AKT and JNK signaling pathways (67, 68). Activation of AKT induces phosphorylation of IκB, which promotes nuclear translocation of NF-κB. JNK also triggers nuclear translocation of AP-1. The expression of ICAM-1 and VCAM-1 in HUVECs is accelerated by the transcription factors AP-1 and NF-κB (52, 53). IL-32θA94V downregulated this signaling pathway by inhibiting TNF-α-induced phosphorylation of FAK, which lies upstream of JNK and AKT, thus attenuating ICAM-1 and VCAM-1 expression (**Figures 7**, **8**).

**Figure 9:**
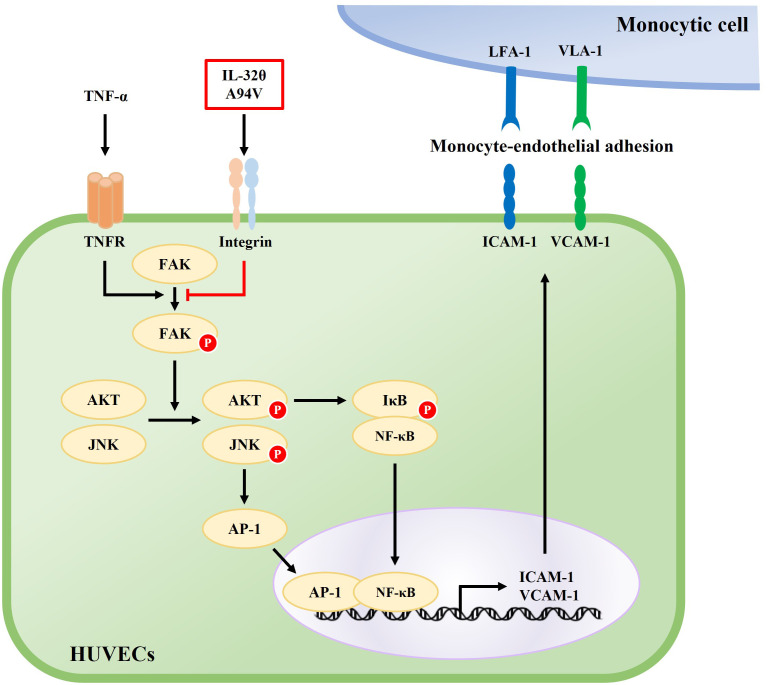
Schematic diagram of the effect of IL-32θA94V on the TNF-α-induced ICAM-1 and VCAM-1 expression signaling pathway. TNF-α-induced phosphorylation of FAK, AKT, JNK, and nuclear translocation of NF-κB, AP-1 promotes the expression of ICAM-1 and VCAM-1. IL-32θA94V inhibits the expression of ICAM-1 and VCAM-1 by inhibiting these intracellular signaling pathways.

Increasing evidence suggests that ICAM-1 and VCAM-1 enhance the inflammatory response and are involved in various diseases (69–72). It has been well known that ICAM-1 and VCAM-1 play roles in arresting immune cells and initiating TEM, which plays an essential step in the development of atherosclerosis. Atherosclerosis, a chronic inflammatory condition triggered by inflammatory cytokines, is the leading cause of most myocardial infarctions and many strokes, leading to high morbidity and mortality (73). Therefore, effective therapeutic strategies targeting ICAM-1 and VCAM-1 can be essential in these diseases. Here, we found that IL-32θA94V mutant attenuated monocyte-endothelial adhesion, a critical early stage in atherosclerosis, by reducing the expression of ICAM-1 and VCAM-1. These results provide a new perspective on the previously known roles of IL-32 in vascular diseases. IL-32θA94V binds to integrins and downregulates the phosphorylation of TNF-α-induced FAK, decreases the phosphorylation of intracellular signaling molecules, such as AKT, JNK, and IκB, and suppresses the nuclear translocation of AP-1 (c-Jun/c-Fos), and NF-κB (p65/p50). Taken together, IL-32θA94V attenuated monocyte-endothelial adhesion by suppressing the expression of ICAM-1 and VCAM-1, which are key factors in atherosclerosis, via integrin-mediated signaling in HUVECs (**Figure 9**). This evidence demonstrates the potential role of IL-32θA94V in the treatment of chronic inflammatory diseases such as atherosclerosis. However, further studies on IL-32θA94V are required using *in vivo* models. Moreover, the possibility of other unidentified cell surface receptors for IL-32 should be investigated. In addition, comparison studies of IL-32θA94V with other isoforms should be performed under various conditions. These studies are important for understanding IL-32θ and its mutant, as well as the overall function of IL-32.

## Data availability statement

The original contributions presented in the study are included in the article/supplementary materials, further inquiries can be directed to the corresponding author/s.

## Author contributions

J-YP and H-MP performed the experiments and wrote the main manuscript text. SK and K-BJ contributed to the analysis and interpretation of the data. C-ML reviewed and revised the manuscript. D-YY and JH supervised the experiments, contributed to the interpreted the results, edited the manuscript. All authors contributed to the article and approved the final submitted version.

## References

[1] Kim SH Han SY Azam T Yoon DY Dinarello CA. Interleukin-32: a cytokine and inducer of tnfalpha. Immunity (2005) 22(1):131–42. doi: 10.1016/j.immuni.2004.12.003 15664165

[2] Joosten LA Netea MG Kim SH Yoon DY Oppers-Walgreen B Radstake TR. Il-32, a proinflammatory cytokine in rheumatoid arthritis. Proc Natl Acad Sci U.S.A. (2006) 103(9):3298–303. doi: 10.1073/pnas.0511233103 PMC1413916 16492735

[3] Heinhuis B Koenders MI van de Loo FA Netea MG van den Berg WB Joosten LA. Inflammation-dependent secretion and splicing of il-32{Gamma} in rheumatoid arthritis. Proc Natl Acad Sci U.S.A. (2011) 108(12):4962–7. doi: 10.1073/pnas.1016005108 PMC3064318 21383200

[4] Netea MG Azam T Ferwerda G Girardin SE Walsh M Park JS. Il-32 synergizes with nucleotide oligomerization domain (Nod) 1 and Nod2 ligands for il-1beta and il-6 production through a caspase 1-dependent mechanism. Proc Natl Acad Sci U.S.A. (2005) 102(45):16309–14. doi: 10.1073/pnas.0508237102 PMC1283464 16260731

[5] Shioya M Nishida A Yagi Y Ogawa A Tsujikawa T Kim-Mitsuyama S. Epithelial overexpression of interleukin-32alpha in inflammatory bowel disease. Clin Exp Immunol (2007) 149(3):480–6. doi: 10.1111/j.1365-2249.2007.03439.x PMC2219317 17590175

[6] Marcondes AM Mhyre AJ Stirewalt DL Kim SH Dinarello CA Deeg HJ. Dysregulation of il-32 in myelodysplastic syndrome and chronic myelomonocytic leukemia modulates apoptosis and impairs nk function. Proc Natl Acad Sci U.S.A. (2008) 105(8):2865–70. doi: 10.1073/pnas.0712391105 PMC2268551 18287021

[7] Sorrentino C Di Carlo E. Expression of il-32 in human lung cancer is related to the histotype and metastatic phenotype. Am J Respir Crit Care Med (2009) 180(8):769–79. doi: 10.1164/rccm.200903-0400OC 19628777

[8] Kang YH Park MY Yoon DY Han SR Lee CI Ji NY. Dysregulation of overexpressed il-32alpha in hepatocellular carcinoma suppresses cell growth and induces apoptosis through inactivation of nf-kappab and bcl-2. Cancer Lett (2012) 318(2):226–33. doi: 10.1016/j.canlet.2011.12.023 22198481

[9] Na SJ So SH Lee KO Choi YC. Elevated serum level of interleukin-32alpha in the patients with myasthenia gravis. J Neurol (2011) 258(10):1865–70. doi: 10.1007/s00415-011-6036-7 21487807

[10] Park JS Choi SY Lee JH Lee M Nam ES Jeong AL. Interleukin-32beta stimulates migration of mda-Mb-231 and mcf-7cells Via the vegf-Stat3 signaling pathway. Cell Oncol (Dordr) (2013) 36(6):493–503. doi: 10.1007/s13402-013-0154-4 24114327 PMC13007480

[11] Dos Santos JC Barroso de Figueiredo AM Teodoro Silva MV Cirovic B de Bree LCJ Damen M. Beta-Glucan-Induced trained immunity protects against leishmania braziliensis infection: a crucial role for il-32. Cell Rep (2019) 28(10):2659–72 e6. doi: 10.1016/j.celrep.2019.08.004 31484076

[12] Moreira Gabriel E Wiche Salinas TR Gosselin A Larouche-Anctil E Durand M Landay AL. Overt il-32 isoform expression at intestinal level during hiv-1 infection is negatively regulated by il-17a. AIDS (2021) 35(12):1881–94. doi: 10.1097/QAD.0000000000002972 PMC8416712 34101628

[13] Goda C Kanaji T Kanaji S Tanaka G Arima K Ohno S. Involvement of il-32 in activation-induced cell death in T cells. Int Immunol (2006) 18(2):233–40. doi: 10.1093/intimm/dxh339 16410314

[14] Kang JW Park YS Lee DH Kim MS Bak Y Ham SY. Interaction network mapping among il-32 isoforms. Biochimie (2014) 101:248–51. doi: 10.1016/j.biochi.2014.01.013 24472437

[15] Shim S Lee S Hisham Y Kim S Nguyen TT Taitt AS. A paradoxical effect of interleukin-32 isoforms on cancer. Front Immunol (2022) 13:837590. doi: 10.3389/fimmu.2022.837590 35281008 PMC8913503

[16] Pham TH Bak Y Kwon T Kwon SB Oh JW Park JH. Interleukin-32theta inhibits tumor-promoting effects of macrophage-secreted Ccl18 in breast cancer. Cell Commun Signal (2019) 17(1):53. doi: 10.1186/s12964-019-0374-y 31126309 PMC6534939

[17] Bak Y Kang JW Kim MS Park YS Kwon T Kim S. Il-32theta downregulates Ccl5 expression through its interaction with pkcdelta and Stat3. Cell Signal (2014) 26(12):3007–15. doi: 10.1016/j.cellsig.2014.09.015 25280942

[18] Park HM Park JY Kim NY Kim JJ Pham TH Hong JT. Modulatory effects of point-mutated Il-32θ on tumor progression in triple-negative breast cancer cells. Biofactors (2023). BIOF-23-0115. 10.1002/biof.2005 37658685

[19] Heinhuis B Koenders MI van den Berg WB Netea MG Dinarello CA Joosten LA. Interleukin 32 (Il-32) contains a typical alpha-helix bundle structure that resembles focal adhesion targeting region of focal adhesion kinase-1. J Biol Chem (2012) 287(8):5733–43. doi: 10.1074/jbc.M111.288290 PMC3285345 22203669

[20] Frank PG Lisanti MP. Icam-1: role in inflammation and in the regulation of vascular permeability. Am J Physiol Heart Circ Physiol (2008) 295(3):H926–H7. doi: 10.1152/ajpheart.00779.2008 PMC2544488 18689494

[21] Kong DH Kim YK Kim MR Jang JH Lee S. Emerging roles of vascular cell adhesion molecule-1 (Vcam-1) in immunological disorders and cancer. Int J Mol Sci (2018) 19(4):1057. doi: 10.3390/ijms19041057 29614819 PMC5979609

[22] Yusuf-Makagiansar H Anderson ME Yakovleva TV Murray JS Siahaan TJ. Inhibition of lfa-1/Icam-1 and vla-4/Vcam-1 as a therapeutic approach to inflammation and autoimmune diseases. Med Res Rev (2002) 22(2):146–67. doi: 10.1002/med.10001 11857637

[23] Stanciu LA Djukanovic R. The role of icam-1 on T-cells in the pathogenesis of asthma. Eur Respir J (1998) 11(4):949–57. doi: 10.1183/09031936.98.11040949 9623703

[24] Veale DJ Maple C. Cell adhesion molecules in rheumatoid arthritis. Drugs Aging (1996) 9(2):87–92. doi: 10.2165/00002512-199609020-00003 8820794

[25] Troncoso MF Ortiz-Quintero J Garrido-Moreno V Sanhueza-Olivares F Guerrero-Moncayo A Chiong M. Vcam-1 as a predictor biomarker in cardiovascular disease. Biochim Biophys Acta Mol Basis Dis (2021) 1867(9):166170. doi: 10.1016/j.bbadis.2021.166170 34000374

[26] Azcutia V Abu-Taha M Romacho T Vazquez-Bella M Matesanz N Luscinskas FW. Inflammation determines the pro-adhesive properties of high extracellular d-glucose in human endothelial cells *in vitro* and rat microvessels *in vivo* . PloS One (2010) 5(4):e10091. doi: 10.1371/journal.pone.0010091 20386708 PMC2851654

[27] Wang Y Cao J Fan Y Xie Y Xu Z Yin Z. Artemisinin inhibits monocyte adhesion to huvecs through the nf-kappab and mapk pathways *in vitro* . Int J Mol Med (2016) 37(6):1567–75. doi: 10.3892/ijmm.2016.2579 PMC4866958 27122190

[28] Vestweber D. How leukocytes cross the vascular endothelium. Nat Rev Immunol (2015) 15(11):692–704. doi: 10.1038/nri3908 26471775

[29] Lefer DJ Granger DN. Monocyte rolling in early atherogenesis: vital role in lesion development. Circ Res (1999) 84(11):1353–5. doi: 10.1161/01.res.84.11.1353 10364573

[30] Lusis AJ. Atherosclerosis. Nature (2000) 407(6801):233–41. doi: 10.1038/35025203 PMC2826222 11001066

[31] Tabas I. 2016 Russell Ross Memorial lecture in vascular biology: molecular-cellular mechanisms in the progression of atherosclerosis. Arterioscler Thromb Vasc Biol (2017) 37(2):183–9. doi: 10.1161/ATVBAHA.116.308036 PMC5269511 27979856

[32] Ross R. Atherosclerosis is an inflammatory disease. Am Heart J (1999) 138(5 Pt 2):S419–20. doi: 10.1016/s0002-8703(99)70266-8 10539839

[33] Frostegard J. Immunity, atherosclerosis and cardiovascular disease. BMC Med (2013) 11:117. doi: 10.1186/1741-7015-11-117 23635324 PMC3658954

[34] Greaves DR Gordon S. Immunity, atherosclerosis and cardiovascular disease. Trends Immunol (2001) 22(4):180–1. doi: 10.1016/s1471-4906(00)01848-2 11394351

[35] Maguire EM Pearce SWA Xiao Q. Foam cell formation: a new target for fighting atherosclerosis and cardiovascular disease. Vascul Pharmacol (2019) 112:54–71. doi: 10.1016/j.vph.2018.08.002 30115528

[36] Heinhuis B Popa CD van Tits BL Kim SH Zeeuwen PL van den Berg WB. Towards a role of interleukin-32 in atherosclerosis. Cytokine (2013) 64(1):433–40. doi: 10.1016/j.cyto.2013.05.002 23727326

[37] Nold-Petry CA Nold MF Zepp JA Kim SH Voelkel NF Dinarello CA. Il-32-Dependent effects of il-1beta on endothelial cell functions. Proc Natl Acad Sci U.S.A. (2009) 106(10):3883–8. doi: 10.1073/pnas.0813334106 PMC2656174 19228941

[38] Kim KH Shim JH Seo EH Cho MC Kang JW Kim SH. Interleukin-32 monoclonal antibodies for immunohistochemistry, Western blotting, and Elisa. J Immunol Methods (2008) 333(1-2):38–50. doi: 10.1016/j.jim.2007.12.017 18252253

[39] Yang J Yan R Roy A Xu D Poisson J Zhang Y. The I-tasser suite: protein structure and function prediction. Nat Methods (2015) 12(1):7–8. doi: 10.1038/nmeth.3213 PMC4428668 25549265

[40] Yang J Zhang Y. I-Tasser server: new development for protein structure and function predictions. Nucleic Acids Res (2015) 43(W1):W174–81. doi: 10.1093/nar/gkv342 PMC4489253 25883148

[41] Zheng W Zhang C Li Y Pearce R Bell EW Zhang Y. Folding non-homologous proteins by coupling deep-learning contact maps with I-tasser assembly simulations. Cell Rep Methods (2021) 1(3):100014. doi: 10.1016/j.crmeth.2021.100014 34355210 PMC8336924

[42] Huang SY Zou X. An iterative knowledge-based scoring function for protein-protein recognition. Proteins (2008) 72(2):557–79. doi: 10.1002/prot.21949 18247354

[43] Huang SY Zou X. A knowledge-based scoring function for protein-rna interactions derived from a statistical mechanics-based iterative method. Nucleic Acids Res (2014) 42(7):e55. doi: 10.1093/nar/gku077 24476917 PMC3985650

[44] Yan Y Wen Z Wang X Huang SY. Addressing recent docking challenges: a hybrid strategy to integrate template-based and free protein-protein docking. Proteins (2017) 85(3):497–512. doi: 10.1002/prot.25234 28026062

[45] Yan Y Zhang D Zhou P Li B Huang SY. Hdock: a web server for protein-protein and protein-DNA/Rna docking based on a hybrid strategy. Nucleic Acids Res (2017) 45(W1):W365–W73. doi: 10.1093/nar/gkx407 PMC5793843 28521030

[46] Yan Y Tao H He J Huang SY. The hdock server for integrated protein-protein docking. Nat Protoc (2020) 15(5):1829–52. doi: 10.1038/s41596-020-0312-x 32269383

[47] Livak KJ Schmittgen TD. Analysis of relative gene expression data using real-time quantitative pcr and the 2(-delta delta C(T)) method. Methods (2001) 25(4):402–8. doi: 10.1006/meth.2001.1262 11846609

[48] Bradford MM. A rapid and sensitive method for the quantitation of microgram quantities of protein utilizing the principle of protein-dye binding. Anal Biochem (1976) 72:248–54. doi: 10.1006/abio.1976.9999 942051

[49] Petersen EJ Miyoshi T Yuan Z Hirohata S Li JZ Shi W. Sirna silencing reveals role of vascular cell adhesion molecule-1 in vascular smooth muscle cell migration. Atherosclerosis (2008) 198(2):301–6. doi: 10.1016/j.atherosclerosis.2007.10.015 PMC2396871 18054358

[50] Ramer R Bublitz K Freimuth N Merkord J Rohde H Haustein M. Cannabidiol inhibits lung cancer cell invasion and metastasis Via intercellular adhesion molecule-1. FASEB J (2012) 26(4):1535–48. doi: 10.1096/fj.11-198184 22198381

[51] Schneider CA Rasband WS Eliceiri KW. Nih image to imagej: 25 years of image analysis. Nat Methods (2012) 9(7):671–5. doi: 10.1038/nmeth.2089 PMC5554542 22930834

[52] Yang CM Luo SF Hsieh HL Chi PL Lin CC Wu CC. Interleukin-1beta induces icam-1 expression enhancing leukocyte adhesion in human rheumatoid arthritis synovial fibroblasts: involvement of erk, jnk, ap-1, and nf-kappab. J Cell Physiol (2010) 224(2):516–26. doi: 10.1002/jcp.22153 20432452

[53] Kim KH Park JK Choi YW Kim YH Lee EN Lee JR. Hexane extract of aged black garlic reduces cell proliferation and attenuates the expression of icam-1 and Vcam−1 in tnf-Alpha-Activated human endometrial stromal cells. Int J Mol Med (2013) 32(1):67–78. doi: 10.3892/ijmm.2013.1362 23619991

[54] Son DJ Jung YY Seo YS Park H Lee DH Kim S. Interleukin-32alpha inhibits endothelial inflammation, vascular smooth muscle cell activation, and atherosclerosis by upregulating Timp3 and reck through suppressing mRNA-205 biogenesis. Theranostics (2017) 7(8):2186–203. doi: 10.7150/thno.18407 PMC5505053 28740544

[55] Lin X Yang L Wang G Zi F Yan H Guo X. Interleukin-32alpha promotes the proliferation of multiple myeloma cells by inducing production of il-6 in bone marrow stromal cells. Oncotarget (2017) 8(54):92841–54. doi: 10.18632/oncotarget.21611 PMC5696226 29190960

[56] Kobayashi H Huang J Ye F Shyr Y Blackwell TS Lin PC. Interleukin-32beta propagates vascular inflammation and exacerbates sepsis in a mouse model. PloS One (2010) 5(3):e9458. doi: 10.1371/journal.pone.0009458 20221440 PMC2832764

[57] Park MH Yoon DY Ban JO Kim DH Lee DH Song S. Decreased severity of collagen antibody and lipopolysaccharide-induced arthritis in human il-32beta overexpressed transgenic mice. Oncotarget (2015) 6(36):38566–77. doi: 10.18632/oncotarget.6160 PMC4770721 26497686

[58] Jung MY Son MH Kim SH Cho D Kim TS. Il-32gamma induces the maturation of dendritic cells with Th1- and Th17-polarizing ability through enhanced il-12 and il-6 production. J Immunol (2011) 186(12):6848–59. doi: 10.4049/jimmunol.1003996 21551364

[59] Son MH Jung MY Choi S Cho D Kim TS. Il-32gamma induces chemotaxis of activated T cells Via dendritic cell-derived Ccl5. Biochem Biophys Res Commun (2014) 450(1):30–5. doi: 10.1016/j.bbrc.2014.05.052 24882804

[60] Kim MS Kang JW Jeon JS Kim JK Kim JW Hong J. Il-32theta gene expression in acute myeloid leukemia suppresses tnf-alpha production. Oncotarget (2015) 6(38):40747–61. doi: 10.18632/oncotarget.5688 PMC4747366 26516703

[61] Bak Y Kwon T Bak IS Hong J Yu DY Yoon DY. Il-32theta inhibits stemness and epithelial-mesenchymal transition of cancer stem cells Via the Stat3 pathway in colon cancer. Oncotarget (2016) 7(6):7307–17. doi: 10.18632/oncotarget.7007 PMC4872787 26824417

[62] Shim S Lee S Hisham Y Kim S Nguyen TT Taitt AS. Comparison of the seven interleukin-32 isoforms' biological activities: il-32theta possesses the most dominant biological activity. Front Immunol (2022) 13:837588. doi: 10.3389/fimmu.2022.837588 35281066 PMC8914309

[63] Hayman EG Pierschbacher MD Suzuki S Ruoslahti E. Vitronectin–a major cell attachment-promoting protein in fetal bovine serum. Exp Cell Res (1985) 160(2):245–58. doi: 10.1016/0014-4827(85)90173-9 2412864

[64] Takada Y Takada YK Fujita M. Crosstalk between insulin-like growth factor (Igf) receptor and integrins through direct integrin binding to Igf1. Cytokine Growth Factor Rev (2017) 34:67–72. doi: 10.1016/j.cytogfr.2017.01.003 28190785 PMC5401657

[65] Barczyk M Carracedo S Gullberg D. Integrins. Cell Tissue Res (2010) 339(1):269–80. doi: 10.1007/s00441-009-0834-6 PMC2784866 19693543

[66] Yoon H Dehart JP Murphy JM Lim ST. Understanding the roles of fak in cancer: inhibitors, genetic models, and new insights. J Histochem Cytochem (2015) 63(2):114–28. doi: 10.1369/0022155414561498 PMC4305513 25380750

[67] Liu S Xu SW Kennedy L Pala D Chen Y Eastwood M. Fak is required for tgfbeta-induced jnk phosphorylation in fibroblasts: implications for acquisition of a matrix-remodeling phenotype. Mol Biol Cell (2007) 18(6):2169–78. doi: 10.1091/mbc.e06-12-1121 PMC1877111 17409352

[68] Oudart JB Doue M Vautrin A Brassart B Sellier C Dupont-Deshorgue A. The anti-tumor Nc1 domain of collagen xix inhibits the fak/ Pi3k/Akt/Mtor signaling pathway through Alphavbeta3 integrin interaction. Oncotarget (2016) 7(2):1516–28. doi: 10.18632/oncotarget.6399 PMC4811477 26621838

[69] Pflugfelder SC Stern M Zhang S Shojaei A. Lfa-1/Icam-1 interaction as a therapeutic target in dry eye disease. J Ocul Pharmacol Ther (2017) 33(1):5–12. doi: 10.1089/jop.2016.0105 27906544 PMC5240001

[70] Kotteas EA Boulas P Gkiozos I Tsagkouli S Tsoukalas G Syrigos KN. The intercellular cell adhesion molecule-1 (Icam-1) in lung cancer: implications for disease progression and prognosis. Anticancer Res (2014) 34(9):4665–72. 25202042

[71] Perkins TN Oczypok EA Milutinovic PS Dutz RE Oury TD. Rage-dependent vcam-1 expression in the lung endothelium mediates il-33-Induced allergic airway inflammation. Allergy (2019) 74(1):89–99. doi: 10.1111/all.13500 29900561

[72] Gao S Yu L Zhang J Li X Zhou J Zeng P. Expression and clinical significance of vcam-1, il-6, and il-17a in patients with allergic rhinitis. Ann Palliat Med (2021) 10(4):4516–22. doi: 10.21037/apm-21-546 33966399

[73] Libby P Buring JE Badimon L Hansson GK Deanfield J Bittencourt MS. Atherosclerosis. Nat Rev Dis Primers (2019) 5(1):56. doi: 10.1038/s41572-019-0106-z 31420554

